# Improving executive functioning and reducing the risk of Alzheimer's disease with music therapy: A narrative review of potential neural mechanisms

**DOI:** 10.1177/13872877251327762

**Published:** 2025-03-24

**Authors:** Benjamin Slade, Ben Williams, Romy Engelbrecht, Joseph Ciorciari

**Affiliations:** 1 Centre for Mental Health and Brain Science, Swinburne University of Technology, John Street Hawthorn VIC, Melbourne, Australia; 2 School of Health Sciences, Swinburne University of Technology, John Street Hawthorn VIC, Melbourne, Australia; 3 Department of Psychological Sciences, Swinburne University of Technology, John Street Hawthorn VIC, Melbourne, Australia

**Keywords:** Alzheimer's disease, executive functioning, health intervention, music training and therapy, neural networks

## Abstract

The incidence of Alzheimer's disease (AD) and the concurrent cost of healthcare will increase as the population continues to age. Pharmaceutical interventions effectively manage symptoms of AD but carry side effects and ineffectively address underlying causes and disease prevention. Non-pharmaceutical interventions for AD, such as music training and therapy do not carry these side effects and can improve symptoms, and should therefore be explored as stand-alone or co-therapy for AD. In addition, music encapsulates modifiable lifestyle factors, such as cognitive stimulation, that have been shown to delay progression of and prevent AD. However, the neural mechanisms underpinning how music improves AD symptoms are not fully understood and whether music can target compensatory processes, activate neural networks, or even slow or prevent AD needs further research. Research suggests neural mechanism may involve stimulating brain areas to promote neurogenesis, dopaminergic rewards systems, and the default mode network (DMN). Alternatively, this review proposes that music improve symptoms of AD via the fronto-parietal control network (FPCN), the salience network (SN) and DMN, and neural compensation. This review will then present evidence for how music could activate the FPCN, SN, and DMN to improve their efficiency, organization, and cognitive functions they govern, protecting the brain from damage, slowing progression, and possibly preventing AD. Establishing how music improves symptoms of AD can lead to tailored music therapy protocols that target functional neural networks responsible for impaired executive functions common in AD.

## Alzheimer's disease, current treatment, and music therapy

The population is ageing, leading to increasing incidences of dementia.^1^ Between 1990 and 2019, the global incidence of dementia increased by 147.5%,^2^ to an estimated 57.4 million cases in 2019. The incidence of dementia is predicted to further increase to an estimated 152.8 million people by 2050.^3^ Alzheimer's disease (AD) is the most common subtype and cause of dementia,^4^ representing between 60% and 70% of all dementia cases.^4^ The incidence of AD also increases with age, in that, for every 1000 people the estimated incidence of AD between ages 65 −74 is 3.43, increasing to 13.78 between 75–84 years, and increasing again to 35.74 over 85 years.^5^

Addressing the increasing incidence of AD is complex as there is no cure, however pharmaceutical interventions effectively manage symptoms.^6^ Currently approved pharmaceutical interventions for AD are acetylcholinesterase inhibitors (donepezil, galantamine, and rivastigmine) and memantine, that targets the N–methyl-D-aspartic receptors (NMDA).^6^ Acetylcholinesterase inhibitors (AChEIs) are the standard for AD treatment.^7^ These inhibitors address the acetylcholine (ACh) hypothesis of AD onset, which suggests a gradual decrease of ACh at the neuronal level, leading to a loss of cholinergic transport at the pre-synaptic terminal and impairments to cognitive functioning.^7^ AChEIs block ACh, maintaining ACh availability^7^ and shows modest improvements in the Alzheimer's Disease Assessment Scale-cognition subscale,^8,9^ and larger effects in global cognitive functioning.^9^ Memantine, another pharmaceutical intervention for AD, is a glutamate receptor NMDA acid receptor antagonist^10^ and is approved for moderate to severe AD.^11^ NMDA receptors are overactive in AD which leads to glutamate hyperactivation, resulting in excitotoxicity and eventually the death of neurons.^12^ Memantine moderates the neurotoxicity of AD by inhibiting over activity of NDMA receptors, reducing glutamate hyperactivation,^10^ and improves the Mini-Mental State Examination and the Alzheimer's Disease Assessment Scale-cognition subscale.^13^

Although AChEIs and memantine address symptoms of AD in the mild, moderate, and severe stages, patients may experience side effects including nausea, anorexia, vomiting, diarrhea, constipation, headaches, confusion, dizziness, and increased somnolence.^14,15^ Aducanumab and lecanemab have also received Food and Drug Administration approval to treat pre and early AD stages,^6^ as accumulating pathology and changing physiology across moderate and severe stages of AD contributed to serious side effects^16^ such as hemorrhaging and edemas.^16^

Although new pharmaceutical treatments and biological targets for AD are in development, the limited number of treatment options and side effects of current pharmaceutical interventions reflect the challenges in treating AD.^17^ These challenges include complex and slow recruitment processes, limited knowledge of precise changes to molecular and biological processes in the brain that cause AD and how these processes change over the course of the disease, and the extended observation time needed to assess if treatment improves or prevents disease.^6,18^

Therefore, alternative, safer, non-pharmaceutical therapies should be explored to improve symptoms of and reduce the incidence of AD.^19,20^ Non-pharmaceutical interventions for AD are mechanistic compared to pharmaceutical approaches, requiring active participation and engagement for the intervention to be effective.^21^ For example, cognitively engaging activities and aerobic exercise may improve impaired executive functions in AD,^22,23^ and when participated throughout life, may reduce the risk of AD up to 64%,^1^ and the risk of cognitive decline.^24^

Music therapy is a non-pharmaceutical intervention for AD that is cost-effective,^25,26^ easily implemented, has shown to improve symptoms of AD,^27^ and can be designed to include interventions that may reduce the incidence of AD (e.g., cognitive engagement and physical exercise).^10,20^ Furthermore, music is still perceived in AD, even into the severe stages of the disease, as the anterior cingulate (ACC) and the pre-supplementary motor area, areas important for musical processing, are preserved,^28^ suggesting music is a viable non-pharmaceutical intervention for AD.

## Music therapy and proposed neural mechanisms in Alzheimer's disease

Music therapy is the purposeful and directed use of music to improve wellbeing and cognitive outcomes. Music therapy facilitates verbal and non-verbal relationships between the music therapist and participants through reminiscence of significant life events using music,^29^ creating songs to express emotions, develop a sense of ownership and completion, and gives participants meaningful opportunities to express their personal identity.^30^

Receptive and active methods are two main approaches used to structure and implement music therapy.^31^ Receptive music therapy involves participants listening to recorded or live music and is often used as a reminiscence tool (e.g., using a favorite song or genre from an earlier period of someone's life) to evoke emotions and reflections over joyful and meaningful autobiographical memories.^32–34^ Active music therapy creates purposeful nonverbal relationships between the music therapist and the participant through musical playing. This relationship is interactive and is maintained through satisfaction and emotions experienced by participants creating music and executing purposeful and meaningful actions.^32,35,36^

Evidence from systematic reviews suggest active and receptive music therapy improves global cognition, executive functioning, and verbal skills in AD.^27,37–41^ However, the effect of these improvements are modest due to heterogeneous methodologies (e.g., components, structure and length of music therapy programs, and measures used) and variability of AD symptoms and severity, a lack of standardization of therapy programs, varied results and conclusions, and small sample numbers.^35,42,43^

Although the number of randomized control trials are increasing,^27^ and neural mechanisms of how music therapy improves AD symptoms have been proposed,^32^ further work is needed to improve music therapy and AD research. This can be achieved by understanding the neural mechanisms of how music therapy improves symptoms of AD, as this would further improve the efficacy of music therapy for AD, strengthen objective evidence for music therapy, and would assist in developing tailored music therapy programs that could address specific symptoms and severities of AD.

As musical processing and engagement requires complex interactions of neural areas and systems, how music therapy improves specific symptoms of AD is not fully understood.^32^ Proposed neural mechanisms of how music therapy generally improves symptoms of AD may involve increasing activity of the limbic system and reward pathways, levels of neurotransmitters, and activity of neural networks.^32^

Emotions evoked by music implicates the limbic and reward systems, including the hippocampus, amygdala, caudate nucleus, auditory cortex, cingulate cortex, pre-supplementary motor area, and the nucleus accumbens.^44,45^ When evoked by music, activation of the limbic system is associated with autobiographical reminiscence,^45,46^ a common feature of music assisted reminiscence therapy.^34,47^ Although the hippocampus is an area of the brain that is first to show AD related pathology and atrophy,^48^ its involvement in music proposes interesting, but speculative mechanisms of how music therapy might work in AD.^32^ Increasing activity of the hippocampus, specifically the dentate nucleus, may lead to neural plasticity and generate new neurons via neurogenesis.^32,49^ Neurogenesis improves cognitive functioning and flexibility, and slows the rate of atrophy in the hippocampus.^50^ Neurogenesis occurs throughout life, but decreases sharply with AD due to pathology in the hippocampus.^51,52^ Whether neurogenesis can be induced to recover cognitive functions in AD is unclear due to limitations of non-invasive approaches to measure molecular mechanisms of neurogensis.^51^ However, animal studies have established that neurogenesis can be enhanced through exercise and cognitive stimulation,^53,54^ which may provide ground work for future human research.

Brain derived neurotropic factor (BDNF), a growth factor important for regulating neuronal differentiation, neuroplasticity neurogenesis, and protecting neurons from cell death in the brain,^55^ facilitates neuroplasticity in the hippocampus.^56^ Hippocampal and serum levels of BDNF are reduced in AD, particularly in later stages of the disease.^57,58^ Serums levels of BDNF increase with donepezil,^59^ and non-pharmaceutical approaches such as exercise and cognitive training.^55,60^ In human research there is no conclusive evidence of the relationship between BDNF, music, and AD, but one possible mechanism is that BDNF expression increases when depression is reduced^61^ and as music therapy reduces depression in AD,^32^ music therapy could boost BDNF production. Animal research shows more conclusive evidence of BDNF increases after exposure to music in AD transgenic mice.^62^

Another potential mechanism by which music therapy may improve symptoms of AD involves the mesolimbic dopaminergic reward pathway.^32^ Music has been shown to activate dopamine reward systems in the brain, particularly when music is liked.^63,64^ Increasing the availability of dopamine in the brain also increases the hedonic response to music, and therefore, suggests how music might improve mood and maintains cognition in AD.^65^

Lastly, the default mode network (DMN) is primarily activated during episodic memory processing, self-reflection, and mind wandering,^66,67^ and is strongly associated with executive function performance.^68^ Activation of the DMN occurs when favorite music is listened to or when music is emotive,^69^ suggesting activation of the DMN via music, can retrieve and re-process episodic and autobiographical memories, which are crucial components of music assisted reminiscence therapy.^47^ The DMN is structurally and functionally impaired in AD and consequently impairs memory and cognition,^70^ but can be targeted by cognitive training, improving executive functioning performance by upregulating activity and compensation of the DMN.^71,72^

Whether emotions evoked by music or music therapy can contribute to the generation of new neurons, increase BDNF, activate reward pathways, or activate the DMN in AD are not fully understood, but could be speculated. Given music can activate the hippocampus, facilitate engagement of executive functioning, reduces depression, elicits feelings of with reward, and increase DMN activity, links between music, stimulation of the dentate nucleus, increasing expression of BDNF to maintain cognitive performance, and the integration of multiple large scale neural networks could be made. However, these potential neural mechanisms are complex and is therefore difficult to conclude how music therapy improves specific symptoms of AD, such as executive functioning, how or if these neural mechanisms occur simultaneously or separately, and whether specific/multiple networks are involved.

This review presents an alternative neural mechanism by which music therapy improves executive functioning impairments in AD. Specifically focusing on how the fronto-parietal, the salience, and the DMN are involved in AD, how music might improve executive functioning via activating these networks, and these neural networks could potentially be targeted with music to reduce the risks of AD via compensation and scaffolding to maintain executive functioning and protect the brain from AD.^73^

## Alzheimer's disease and functional neural networks

In AD, functional neural networks show continual dysfunctional change in response to neuropathology.^74^ The integrity and segregation of functional neural networks decreases as AD neuropathology increases, leading to behavioral and cognitive impairments characteristic of AD such as agitation, and episodic and working memory.^75–77^ This begins in the prodromal stages of the disease, mild cognitive impairment (MCI), as neural networks show inter-network synchronization.^78^ This leads to compensatory neural activity to maintain higher cortical functions. Increased inter-network compensation is evident within the fronto-parietal control network (FPCN) and the salience network (SN), to compensate for decreased inter-network activation of the DMN.^79,80^

Activity of these functional neural network becomes inefficient and disorganized in the early stages of AD. Increased activity is often observed in the SN, which is correlated with decreased DMN activity^79^ possibly resulting in behavioral responses associated with the SN becoming overly sensitive, which could account for at least some of the increased agitation and aggression often observed in AD patients.^81,82^ The increased activation of the SN in the early stages of the disease could be compensating for the decreased activation in the DMN.^83^ However, as AD progresses to mild and moderate stages, all neural networks show decreased inter- and intra- network activation and consequently, the compensatory activation of the SN on the DMN is lost.^84–86^

The DMN is highly studied within the general AD literature,^87^ as activation occurs during episodic memory recall—a function commonly impaired in AD—and areas within the network are primary sites for amyloid-β deposits.^84^ Increased amyloid-β burden in the DMN suggests increased synaptic dysfunction, decreased cognitive functioning and functional connectivity between other neural networks,^88^ particularly from the precuneus to the hippocampus, the precuneus to the parahippocampus, and precuneus to the anterior cingulate.^89,90^ Functional connectivity changes between the precuneus and the hippocampus in particular, are associated with accelerated neural atrophy, suggesting functional connectivity changes within resting state networks can be used as biomarkers for early cognitive decline and a marker for MCI to AD conversion.^91,92^

Although metabolic decreases are found in AD patients in several resting state networks (the dorsal attention network, the SN, the control network and the sensory motor network^36^), the central sites for decreased connectivity in AD are found in DMN, primarily in the posterior parietal cortex (PCC),^67,82,87^ MTL and the inferior parietal lobe.^93^Furthermore, connectivity and metabolic activity from the PCC to hippocampus and the PCC to the whole brain gradually decreases with disease progression.^93,94^

AD has been described as a “disconnection syndrome”, as neural networks gradually show widespread inter- and intra- network dysfunction (i.e., inefficient neural network connectivity) leading to reduced inter network connectivity and random connectivity patterns.^95–97^ Neural network patterns in AD show greater randomization of connectivity patterns and segregation of networks compared to MCI,^98^ as the shortest path between nodes (a reflection of the minimal number of connections that have to be crossed when traveling between nodes) increases, suggesting decreased global network efficiency^99^ and dysfunctional between hubs.^95^ Hubs of neural networks are also impacted in AD, showing increased levels of amyloid-β deposits, higher levels of atrophy within a network, and are connected to areas that have the greatest atrophy.^97^ The segregation of neural networks progresses and leads to cognitive functions related to the posterior areas of the brain such as episodic memory retrieval and visuospatial functioning.^79^ These cognitive deficits are associated with the hypometabolism and atrophy of the DMN, particularly in the posterior regions of the network.^100^

Neuroimaging evidence suggests that in response to neural network dysfunction, hypoactivation occurs within a neural network and hyperactivation occurs between neural networks.^88,101^ The hypoactivation is reflective of the slow disorganization of a neural network from amyloid-β and tau accumulation, leading to the behavioral and cognitive symptoms of AD. The hyperactivation of the neural networks may reflect early alterations and disorganization of neural networks due to the disease, and possible compensation for the dysfunctional connectivity patterns of neural networks.^88,101^ Posterior areas of the AD brain, particularly areas within the DMN, show the greatest atypical network activation: hypo-activity within the DMN^102^ and hyper-synchronized connectivity between the DMN and other neural networks. Hyper-synchronized connectivity of neural networks suggests a compensatory response^103^ to the neuropathology and synaptic loss in AD.^104^ Network compensation can also be impeded by accumulating neuropathology and neural atrophy, as neural networks become less synchronized and more disorganised.^95^

## Executive functioning, neural networks, and Alzheimer's disease

Executive functioning processes, such as working memory, are distributed across several brain areas including parietal, sensory, temporal, and prefrontal cortices.^105,106^ These cognitive processes rely on communication within and between three large scale brain networks—FPCN, SN, and the DMN—to process sensory information for behavior, and to support and maintain working memory and executive functioning.^105,107^ AD often results in impairments to executive functioning, working memory, and attentional control systems,^108^ specifically for activities such as holding and manipulating visual and auditory information,^109^ carrying out instructions, processing sequential stimuli, switching tasks, and when continuous attention tasks demand different cognitive loads.^108^ Impaired executive function in AD suggest dysfunctional and disorganized activity of FPCN, SN, and the DMN.^10^5

The FPCN is a core working memory network due to its involvement in task switching, task maintenance, goal-directed behavior, and coping with non-routine task demands.^106^ The FPCN prioritizes and coordinates information flow across multiple networks, including the DMN,^110^ and transfers task components such as task rules, knowledge, and skills, from previous tasks to novel tasks.^111^ The FPCN also becomes dysfunctional in AD, and its operation during working memory tasks activation in frontal areas decreases while activation of the posterior parietal cortex increases.^112,113^ This pattern of activation during working memory tasks occurs in response to dysfunctional patterns in other neural networks, as the brain engages alternative pathways (inferior partial lobule, the medial frontal gyrus, and the left inferior frontal gyrus) to maintain cognitive performance.^114^

The SN is a flexible control network controlling aspects of goal directed behavior, and engages the FPCN during executive functioning tasks when salient stimuli are detected.^115–117^ This system is disrupted in AD as the SN becomes dysfunctional with increasing neuropathology. Activity within the SN and between the SN and the locus coeruleus (LC) decreases,^82,118^ resulting in weaker signaling to activate the FPCN and deactivate the DMN.^107^ This leads to disrupted bottom-up processing of salient events and atypical communication between the FPCN and DMN, depleting cognitive resources, increasing cognitive fatigue,^107^ and disrupting maintenance of executive functions.^119^

During working memory tasks, neural resources are diverted from the DMN to task-relevant processes and neural networks, deactivating the DMN.^120,121^ With increasing working memory load, the rate of DMN deactivation is slower in healthy older people compared to healthy adults, suggesting possible compensation.^121^ The inability of the DMN to deactivate in healthy older adults during cognitively demanding tasks reflects lapses in attention, and increasing the activation of the FPCN and SN, suggesting further compensatory processes to maintain cognitive performance.^122^

Neuroimaging evidence suggests targeting neural networks that show compensatory activity in response to non-pharmaceutical interventions, can improve working memory in MCI.^91^ There is little research investigating whether increasing neural network activity with music therapy can improve cognitive functioning in AD, despite music therapy engaging cognitive functions such as conflict monitoring, inhibitory control and working memory,^123^ cognitive functions the FPCN govern and support.^111,124^ As the FCPN shows compensatory activity in older adults and mild AD,^114^ and music is processed via the hub of the SN,^28^ music therapy could be used to enhance activation of these networks to improve and maintain cognitive functions for people with AD.

## Potential mechanisms of how music improves executive functioning in Alzheimer's disease?

Cognitive interventions that improve working memory by targeting the FPCN suggest the involvement of cognitive reserve.^125^ When activated, the FPCN recruits additional neural resources to a task,^126^ or increases compensatory activity to maintain cognitive functioning.^127^ Increasing neural compensation in areas of the FPCN has been shown with AD participants across working memory, executive functioning, attention, and episodic memory^76,77^ and may lead to recruitment of neural areas that are underactive or not directly associated with the task to maintain cognitive function.^128^

In AD, performance on executive functioning tasks (e.g., the n-back task) can be improve with training or non-pharmaceutical interventions that target the neural networks responsible for impaired executive functioning.^91,129^ Cognitive interventions for AD aim to improve encoding and recall, conversation skills, problem solving, creative activities, visuospatial tasks, and task repetition, show moderate to large effect sizes.^130^ Learning how to play a musical instrument requires these cognitive processes and has shown transfer effects to other cognitive tasks unrelated to music^131^ for older adults,^132^ and improves performance on the Mini-Mental State Examination.^133,134^

Participants with AD can recall more novel lyrics compared to novel words,^135^ can recognize and sing to a melody when prompted compared to spoken words,^136^ and can recall novel songs that are sung compared to the same songs when spoken.^137^ Neuroimaging evidence suggest that after a 6-month karaoke training program with AD participants, psychomotor speed and AD symptoms improved, and these improvements were associated with decreased activation of the angular and the left lingual gyrus.^138^ The neuroimaging results suggest musical training facilitates different cognitive strategies and can change the organization and efficiency of neural activation for people with AD.^139^

**Figure 1.**
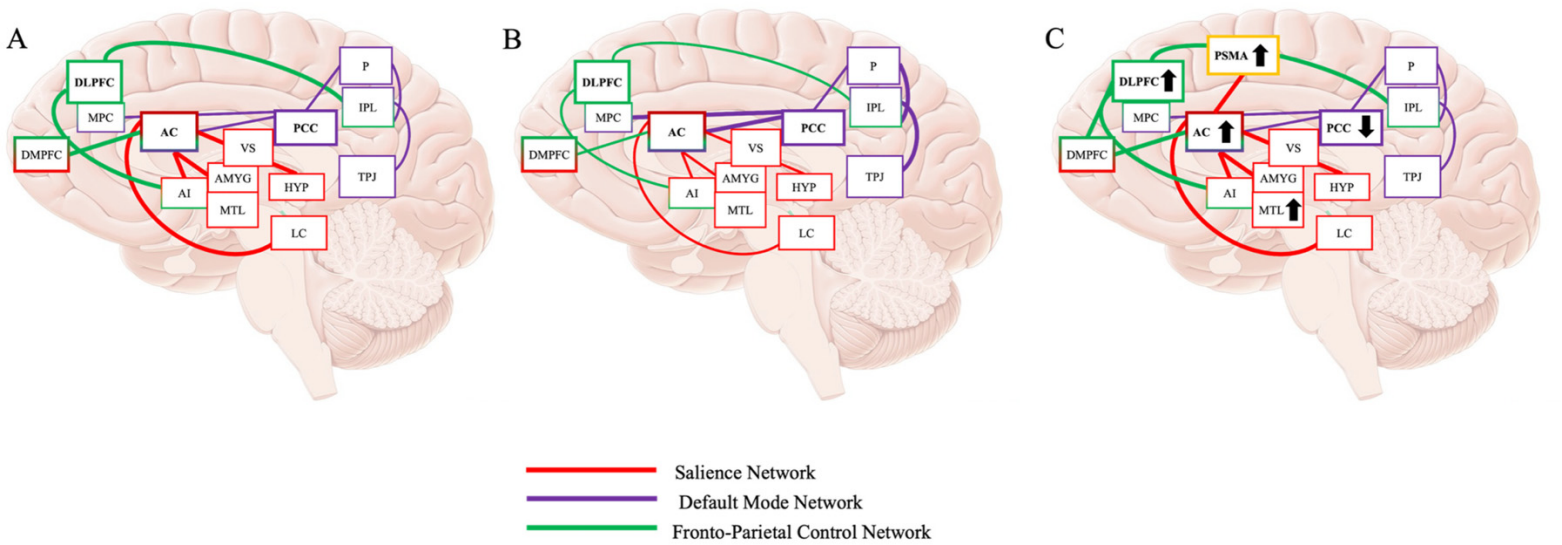
(A) Typical activity of the increased FPCN and SN and decreased DMN during completion of executive functioning tasks. Fronto-Parietal Control Network (Green): DLPFC: dorsal lateral prefrontal cortex; MPC: medial prefrontal cortex; DMPFC: dorsal medial prefrontal cortex; Salience Network (Red): AC: anterior cingulate cortex; AI: anterior insula; VS: ventral striatum; AMYG: amygdala; MTL: medial temporal lobe; HYP: hypothalamus; LC: locus coeruleus; Default Mode Network (Purple): PCC: posterior cingulate cortex; TPJ: temporal parietal junction; IPL: inferior parietal lobule; P: precuneus. (B) Activity of the FPCN and SN and DMN during completion of executive functioning tasks in AD. The SN and FPCN do not increase activation, and DMN does not disengage, leading to impaired processing and performance of executive functioning tasks. (C) Proposed neural mechanisms of how music might improve executive functioning in AD. Increased SN activity occurs via the pre-supplementary motor area (SPMA), which may support FPCN function while DMN decreases, overall improving executive functioning.

In healthy brains, music is perceived in many neural areas including the temporal lobes and hippocampus.^28^ Despite temporal lobe and hippocampal dysfunction and atrophy, people with AD can still perceive, engage, and reminisce with music^35^ suggesting music is perceived via different neural pathways and areas of the brain,^138^ and is further supported by music-based reminiscence therapy for people with AD.^1,33^ The pre-supplementary motor area and anterior cingulate gyrus (ACG) are critical for music perception, storing musical information in long term memory, are preserved in moderate to severe stages of AD, and when compared to areas of the temporal lobe, show less neural atrophy and amyloid-β.^28,32,140^ As the ACG connects to the pre-supplementary motor area when responding to stimuli,^115^ and is the functional hub of the SN,^107,117^ music therapy could target the ACG to increase the activation of the SN, increasing activation of the FPCN, improving working memory and executive functioning.^127^ This evidence presents a hypotheses of how music therapy can be used or designed to address impairments to executive functioning in AD. Based on the presented evidence, we present an alternative neural mechanism for how music therapy might improve symptoms of executive function in AD (Figure 1). We propose, from the auditory cortex, the pre-supplementary motor area and ACC process the components of music. Depending on the type of music, other neural areas, such as the medial temporal lobes and the dorsal lateral prefrontal cortex may also be recruited to support neural networks and maintain executive functioning. As the ACC is the hub of the SN, this may increase activity of the SN, may therefore increase activity of areas within the FPCN, and may also decrease activity of the DMN to improve impaired executive functioning.

## Potential of music to reduce the risk of Alzheimer's disease

Several modifiable lifestyle factors increase (e.g., physical inactivity, depression, smoking) and decrease (e.g., improving levels of physical activity, cognitive engagement, and socialization) AD risk, accounting for approximately 40% of all AD cases worldwide.^141^ In addition, longitudinal evidence suggests participation in lifestyle factors that decrease AD risk, slow rates of cognitive decline and can help sustain performance on executive functioning activities longer than those who are not as well educated and do not seek cognitive activity.^24^ Decreasing the risk of developing AD seems to require active participation and cognitive engagement of lifestyle factors (e.g., physical exercise, cognitive engagement), which facilitates neural plasticity and neural scaffolding, therefore protecting the brain from cognitive decline and AD.^21^ This suggests actively engaging and participating in musical training and therapy (e.g., singing, playing instruments, learning lyrical sequences, and manipulating musical components of songs) is more beneficial to the brain than passive engagement (e.g., listening to music).

Theories of how lifestyle factors protect the brain from cognitive decline and AD, such as the Scaffolding Theory of Ageing and Cognition (STAC-r)^127^ are centered on protecting the brain against damage and pathology.^142^ The STAC-r^127^ proposes that the brain develops neural scaffolding over a lifetime in response to cognitive challenges, acquisition of cognitive skills, and by maintaining social, behavioral, and cognitive performance in response to declining functions from chronological ageing. In support of this hypotheses, long-term musical making may lead to younger appearing brains when compared to the same participants chronological age.^143^ This finding was evident in amateur and professional musicians, but was more pronounced in the amateur musician group, suggesting when music making is seen as an extracurricular past-time and not as a profession, amateur musicians can capitalize on the plasticity and potential protective effects enhanced by music making.^143^

Neural scaffolding provides the brain with supplementary, complementary, and alternative ways to process information in response to behavioral or cognitive demands.^144^ Physical exercise, cognitive enrichment, novel environments, and learning can strengthen neural scaffolding, protecting against dysfunctional activity of neural networks, neural atrophy, and white matter deterioration.^127,145^ Physical exercise, cognitive enrichment, and learning are part of music training and can be easily implemented as part of musical therapy programs, making musical therapy a possible intervention for enhancing neural scaffolding and protecting the brain from age related cognitive decline and AD.

In AD, fronto-parietal regions are recruited by the PCC in response to dysfunctional network connectivity between and within parietal and DMN regions.^127^ The recruitment of compensatory mechanisms enhances neural activity and connectivity, however as the disease progresses, amyloid-β accumulates in areas of higher connectivity^88^ eventually permeating the established scaffolding and collapsing neural compensatory structures.^146^ Strengthening the scaffolding of the fronto-parietal network before the onset of cognitive decline and AD with exercise and cognitive training has shown to improve episodic and semantic memory and may preserve cognition and promote cognitive stability in MCI and AD.^91,147^ Although early participation in lifestyle factors that reduce the incidence and progression of AD are the most effective at protecting the brain, meta-analyses suggest increased efficiency of functional activity in the FPCN and DMN occurs after cognitive training and may reverse compensatory mechanisms while maintaining cognitive performance.^75^ In addition, cognitive training may also increase segregation between functional neural networks in ageing and AD,^75^ which is a measure of cognitive resilience to AD pathology,^146^ indicating better cognitive functioning.^147^

To our knowledge, there no direct evidence musical therapy has be used to increase neural scaffolding and target compensatory mechanisms of neural networks to protect or reduce the incidence of AD. Working towards confirming these hypotheses is also confounded by the limitations of music therapy and AD research (modest effect sizes, varied results and conclusions, and small sample numbers).^35,42,43^ However, given the evidence of neural scaffolding and compensation in AD, musical training and therapy would need to be practiced over a longer period of time and potentially before disease onset.

There is research related to this area, singing training can improve neural efficiency in AD, suggesting the associated neural processes and pathways used to learn how to sing, gradually become more efficient with training,^138^ suggesting music therapy could contribute to neural scaffolding and compensation. However, it may also be the case the music therapy is activating other neural mechanisms such as other areas of the brain that support neural network function to support and maintain cognition.^102,142^

## Conclusion

As a therapy for AD, music address some of the limitations of pharmaceutical approaches and has shown to improve some symptoms of AD, including impaired executive functions. However, definitive conclusions about the benefits of music therapy for AD are limited by inconsistent methods and findings, and have contributed to the limited knowledge of the neural mechanisms of how music therapy might improve symptoms of AD. This review presents evidence for how music training and therapy might improve executive function deficits in AD and how the FPCN, SN, and DMN have significant roles in maintaining cognitive performance throughout ageing, cognitive decline, and AD, and may be therapeutic targets using music. Evidence suggests that music therapy encapsulates modifiable factors that enhance neural scaffolding and target compensational processes in the brain. To understand the mechanisms by which music therapy improves executive functioning or could reduce the risk of AD, future research should ensure music therapy facilitates active participation, is interdisciplinary, and include or consider neuroimaging to objectively assess changes to functional and compensatory activity in neural networks in response to music therapy in AD. Interdisciplinary research can further assess the effectiveness of music in slowing the rate of cognitive decline and AD onset and progression. As music can still be processed, perceived and interacted with during MCI and all stages of AD, in neural areas fundamental to neural networks highly linked with neural compensation, music training and therapy is a viable therapeutic or co-therapeutic modality to improve executive functioning AD.
